# Radiological Lung Sequelae of Severe COVID-19: A Retrospective Observational Study From a Dedicated COVID Centre of Eastern India

**DOI:** 10.7759/cureus.21416

**Published:** 2022-01-19

**Authors:** Deependra Rai, Subhash Kumar, Sanjay Pandey, Harsh Vardhan

**Affiliations:** 1 Pulmonary Medicine, All India Institute of Medical Sciences Patna, Patna, IND; 2 Radiodiagnosis, All India Institute of Medical Sciences Patna, Patna, IND; 3 Physical Medicine and Rehabilitation, All India Institute of Medical Sciences Patna, Patna, IND; 4 Nephrology, All India Institute of Medical Sciences Patna, Patna, IND

**Keywords:** severe acute respiratory syndrome coronavirus 2, pulmonary fibrosis', ct scan chest, post-covid sequelae, covid-19 india

## Abstract

Background: The pulmonary sequelae of severe COVID-19 infection are yet to be fully defined. The authors undertook this study to find out the proportion of severe COVID-19 patients having fibrosis-like lung sequelae during a medium-term follow-up period.

Materials and methods: This was a retrospective observational study from a dedicated COVID centre of Eastern India. Severe COVID-19 patients who had undergone chest computerized tomography (CT) during the acute phase of illness and at least one follow-up CT with a gap of minimum two months between the two scans were included in the study.

Result: A total of 39 patients who had recovered from severe COVID-19 pneumonia and presented to the pulmonary medicine OPD in the months of July and August 2021 were included. Patients with pre-existing lung disease (n-4), mild to moderate (n-11), and due to unavailability of CT scan (n-2) were excluded. A total of 22 patients (thirteen males, nine females) were thus included for analysis. Follow-up scans were performed with a mean of 2.5 months after the onset of the disease. Out of 22 patients, only one patient’s follow-up scan was normal. Predominant fibrotic-like features were present in six (27.2%) patients, though some evidence of fibrosis-like changes were seen in 20 out of 22 (90.9%) patients. The remaining 15 (68.2%) patients with abnormal scans had predominant non-fibrotic changes like ground-glass opacities (GGOs), consolidation, cavity, or nodule. The most common presenting symptoms at the follow-up examination were dyspnoea (81.8%), cough (54.1%) followed by fatigue in 40.9% of patients.

Conclusion: This study concluded that most of the severe COVID-19 patients have some residual radiological findings during medium-term follow-up. Fibrotic-like lesions are present in almost all patients but most of them get resolved with time. True fibrotic features like honeycombing are rarely seen as residual lung sequelae.

## Introduction

Long COVID-19, also known as post-acute sequelae of SARS-CoV-2 infection (PASC) or post COVID-19 condition, is defined as a post-viral syndrome affecting people who have recovered from COVID-19 infection. It can manifest weeks or months after the apparent clinical resolution of the acute phase of the disease. Dyspnoea and fatigue are among the most common symptoms [1-2]. Post COVID-19 sequelae may vary from mild symptoms as fatigue and body ache to severe symptoms that may due to lung fibrosis, cardiac abnormalities, and stroke leading to significant impairment in quality of health [3]. Pulmonary sequelae are one of the long-term consequences of COVID-19 observed in nearly one-third of patients [4].

Chest CT plays a crucial role in the diagnosis and follow-up of patients with COVID-19 infection. Radiologically, pulmonary sequelae can have fibrosis-like manifestations such as reticular opacities, honeycombing, traction bronchiectasis, parenchymal bands as well as non-fibrotic features such as ground-glass opacities (GGOs), consolidation or nodules, etc. We may have some insight from the severe acute respiratory syndrome (SARS) outbreak of 2002-2003 caused by SARS-CoV and the Middle East respiratory syndrome (MERS) first identified in 2012 but literature for the SARS-Cov 2 pulmonary sequelae and its course is sparse [5-6].

The primary objective of the present study is to find out the proportion of patients with fibrosis-like lung sequelae in patients who have recovered from severe COVID-19 infection.

## Materials and methods

The study was performed at a government-designated dedicated COVID-19 center of excellence in Eastern India. All the severe COVID-19 patients followed up at the pulmonary medicine outpatient during July-Aug 2021 were included in the study. Real-time polymerase chain reaction (RT-PCR) or rapid antigen test (RAT) positive severe COVID-19 patients who had undergone high-resolution chest CT (HRCT) during the acute phase of illness and at least one follow-up HRCT with a gap of at least two months between the two scans were included in the study. The relevant demographic characteristics, clinical history, and chest CT findings were collected and analyzed retrospectively.

The following group of patients were excluded: 1. Patient diagnosis based on clinical and radiological features; 2. Follow-up CT available within two months of diagnosis; 3. Patients with pre-existing lung diseases like Interstitial lung disease (ILD), tuberculosis, bronchiectasis.

Case definition

Fibrotic-like sequelae: HRCT showing predominance of radiographic findings suggestive of fibrotic-like features, including either or all of the following - honeycombing, reticular opacities, parenchymal band(s), and traction bronchiectasis.

Non-fibrotic sequelae: HRCT showing predominance of findings other than above, such as GGOs, consolidation, cavities, etc. Such patients, however, may have fibrotic-like HRCT findings as a minor component.

CT image interpretation

HRCT was performed on a 128-slice Philips Ingenuity CT scan system (Philips Healthcare, Best, Netherlands). All CT images were reviewed by a senior radiologist and a pulmonologist, each with more than 10 years of experience; the findings were recorded in consensus. For each patient, the predominant CT patterns according to the Fleischner Society glossary were enumerated [7]. To quantify the extent of pulmonary abnormalities (total lesions, GGO, consolidation, reticulation, and fibrotic-like changes), a semiquantitative CT score was assigned on the basis of the area involved in each of the five lung lobes.

Each lobe of the lung was examined to get a CT severity score. The CT severity score was assessed based on the percentage of involvement of each lobe and then added together: 0- no involvement, 1- less than 5% involvement, 2- 5%-25% involvement, 3- 26%-49% involvement, 4- 50%-75% involvement, 5- Greater than 75% involvement. The total CT severity score was calculated by summing the individual lobar scores, with possible scores ranging from 0 to 25.

Statistical analysis

Data was analysed using Microsoft Excel 2013 and SPSS 22.0 (SPSS Inc., Chicago, IL, USA). Continuous variables were expressed as means and standard deviations. Categorical variables were expressed as counts and percentages. A P-value less than 0.05 was considered statistically significant.

## Results

**Figure 1:**
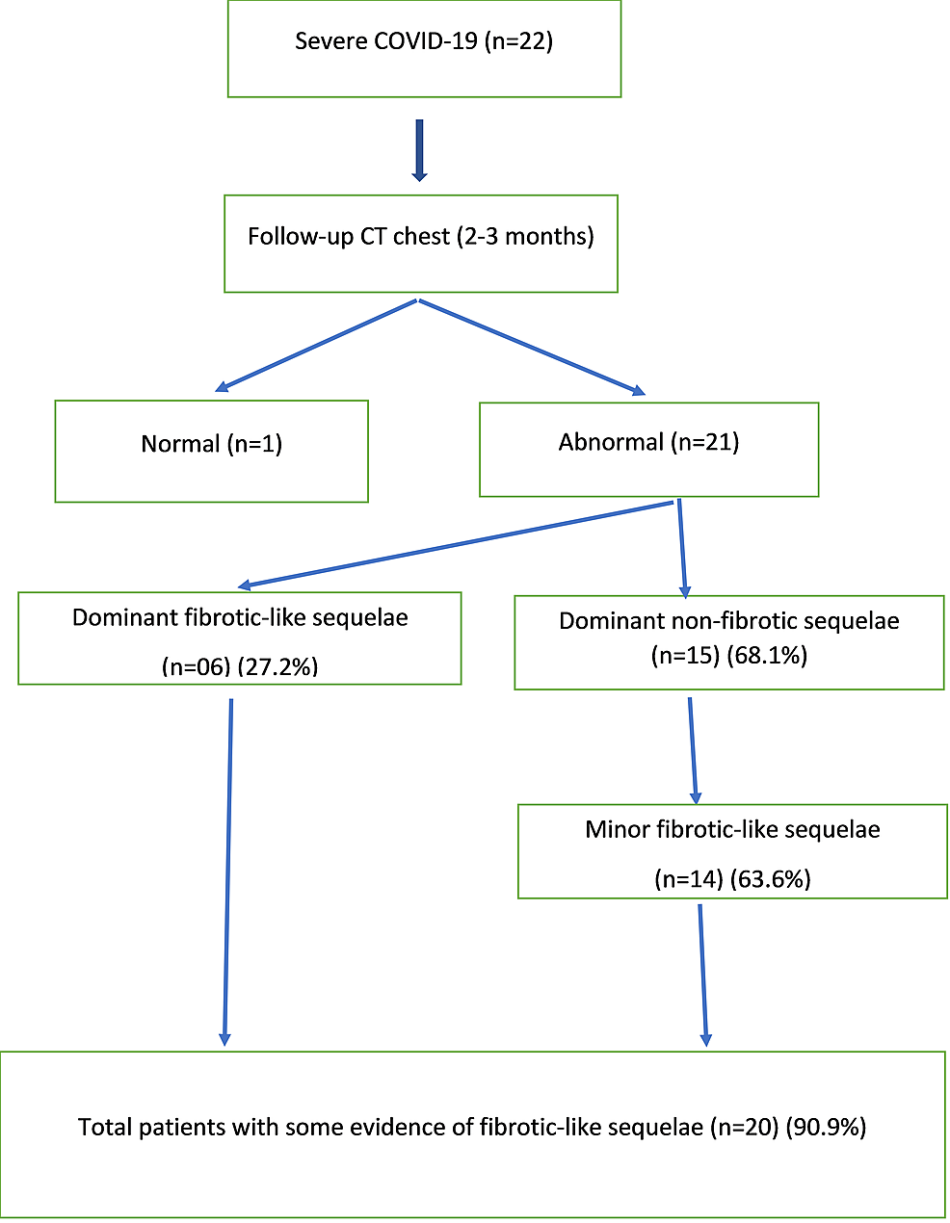
Type of lung sequelae in study patients

A total of 39 patients had presented in the month of July-Aug 2021, who had recovered from severe COVID-19. Patients with pre-existing lung disease (n=4), mild to moderate infection (n=11), and unavailability of CT scans (n=2) were excluded. A total of 22 patients (thirteen males, nine females) were thus analyzed. Follow-up scans were performed with a mean duration of 2.5 months after the disease onset. Out of 22 patients, only one patient’s follow-up scan was normal. Predominant fibrotic-like sequelae were present in six (27.2%) patients and 15 (68.1%) patients had predominantly non-fibrotic-like sequelae, like GGOs, consolidation, cavity, or nodule (Figure 1) (Table 1). However, some evidence of minor fibrotic-like sequelae were seen in 14 out of these 15 cases also, i.e., there was evidence of fibrotic-like radiographic features in 20 out of 22 (90.9%) patients in all, whether minor or major.

**Table 1:** Clinico-demographic characteristics and radiological lung sequelae in study patients

Characteristics	Number (n = 22)	Proportion (100%)
Age (years) (Mean ± SD)	50 ±13.6	-
Male	17	77.2
Presence of Diabetes	07	31.8
Presence of Hypertension	05	22.7
Post Covid Symptoms		
Dyspnoea	18	81.8
Cough	12	54.5
Sputum production	4	18.1
Chest Pain	02	9.0
Fatigue	09	40.9
Throat irritation	05	22.7
Follow-up CT chest (months) (Mean ± SD)	2.59 ± 0.43	-
Normal	01	4.5
Abnormal	21	95.4
Bilateral changes	21	95.4
Dominant pattern		
Fibrotic-like sequelae	06	27.2
Non-fibrotic sequelae	15	68.1
CT Severity Score (CTSS) ((Mean ± SD))	15.25 ± 7.59	-
CT Features		
Ground glass opacity (GGO)	20	90.9
Consolidation	07	31.8
Reticulation	19	86.3
Parenchymal band(s)	20	90.9
Honeycombing	02	9.09
Traction bronchiectasis	15	68.1
Bronchial dilatation	09	40.9
Architectural distortion	14	63.3
Pneumatocele	03	13.6
Cavity	06	27.2
Nodule	01	4.54
Pneumothorax	02	9.0

**Figure 2:**
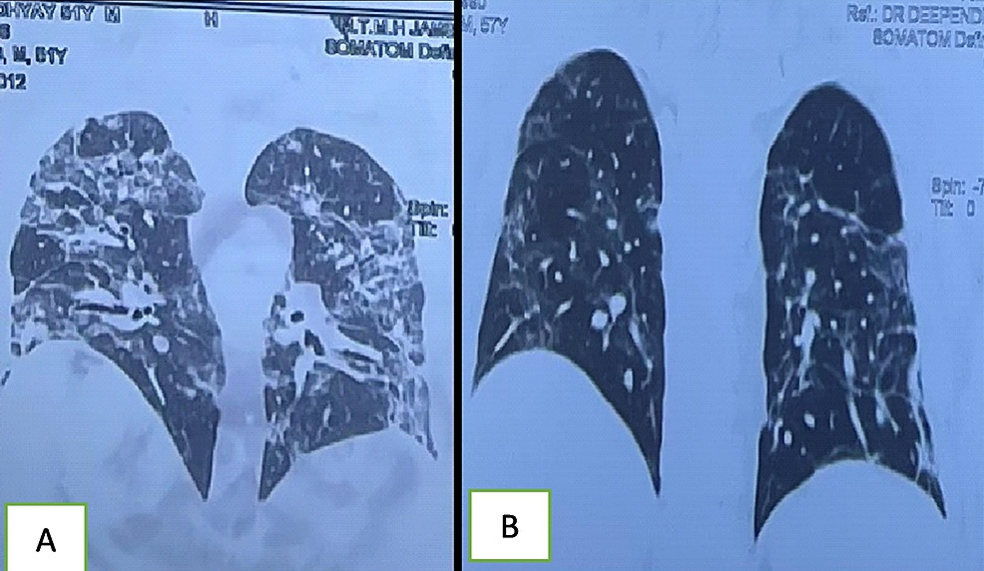
A 60-year-old male with severe COVID-19 Initial CT scan showing predominant ground-glass opacities (GGOs), septal thickening, reticular shadow (A). Follow-up CT three months later showed complete resolution with air trapping in the left upper lobe with reticular shadow (B).

**Figure 3:**
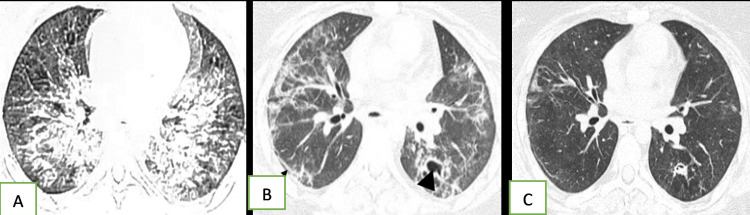
A 23-year-old female with severe COVID-19 Initial CT shows extensive bilateral consolidation and ground-glass opacities (GGOs), predominantly in central distribution (A). The first follow-up CT after 34 days of diagnosis shows mixed reticular and GGOs, as well as parenchymal bands (thin black arrow) mainly in peripheral distribution; an irregular cavity has formed in the left lower lobe (short black arrow) (B). Second follow-up CT after 85 days of diagnosis shows significant resolution of all opacities, as well as reduction in the cavity size (C).

**Figure 4:**
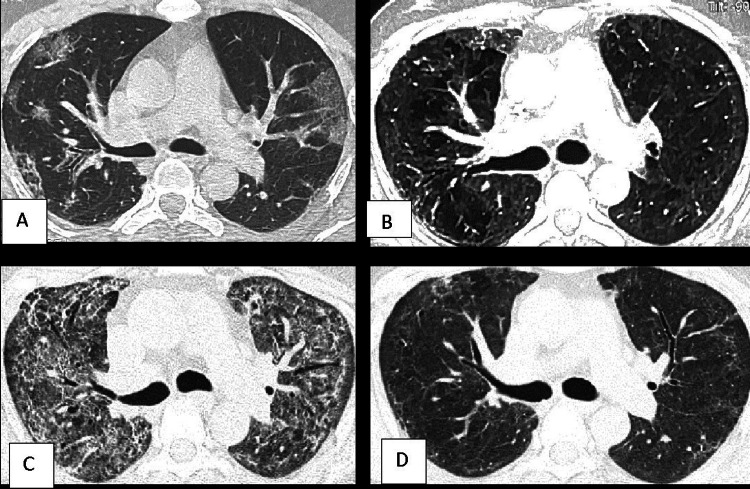
A 40-year-old male with waxing-waning lung CT opacities CT at initial diagnosis of COVID-19 shows geographically shaped, mainly peripheral, ground-glass opacities (GGOs) in both lungs (A). One and a half months later, CT shows a decrease in the geographic lesion, but a mild diffuse haziness throughout the lung parenchyma and few small reticular opacities are evident (B). Two and a half months later, significantly increased ground glass densities, bronchial dilatation, and reticular opacities are seen (C). And four and a half months later, there is a significant resolution of the density of the opacities, however, subtle diffuse haziness persists; note the walls of the earlier dilated bronchi are again smooth without the ‘wavy’ contour (D).

**Figure 5:**
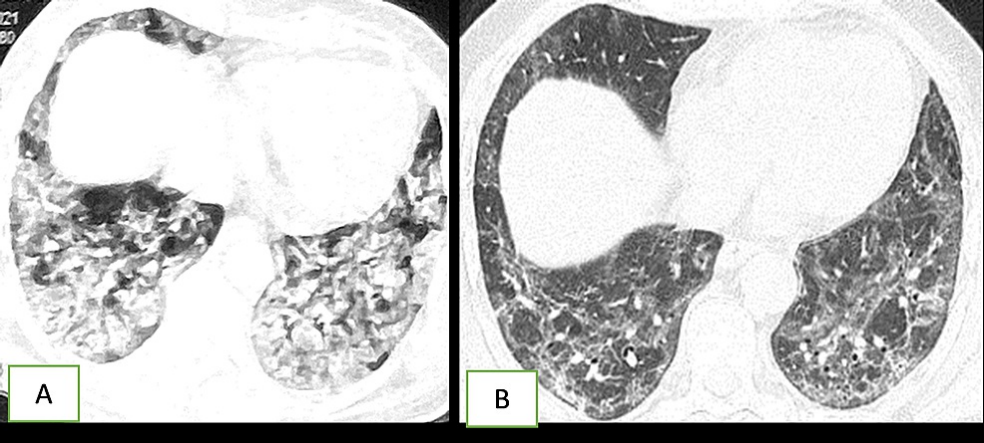
A 59-year-old male with severe COVID-19 Initial CT shows extensive consolidation and some ground-glass opacities in both lungs (A). Follow-up CT at two months shows evolution of the changes, the consolidation has disappeared, fine reticulations producing a diffuse geographical pattern of opacities, as well prominent bronchi are visible in both lungs, intervening lung areas between the geographical opacities and reticulations appear normal (B).

The radiological findings were reduced during follow-up and the residual lesions present were mostly GGOs, reticular, or band-like opacities (Figures 2-5). One patient developed a cavity during follow-up, which subsided with treatment (Figure 3). Another patient developed worsening of radiological findings after initial improvement, and with subsequent improvement (Figure 4). The most common presenting symptoms at the follow-up examination were dyspnoea (81.8%), cough (54.1%), and fatigue in 40.9%.

## Discussion

In this study, we analysed radiological lung sequelae in 22 COVID-19 patients who had recovered from severe disease. Acute COVID-19 pneumonia classically presents radiologically as peripheral, bilateral, diffuse GGOs, consolidation, reticulations, or mixed pattern of lesions [8]. During follow-up, some of these abnormalities persist and are labelled as lung sequelae. Fibrotic-like sequelae, predominantly seen in patients with severe disease, require intensive care unit care [9]. In our series, with the mean follow-up CT performed 2.5 months after the acute phase, only one patient showed complete resolution. Predominant fibrotic-like features were present in six (27.2%) patients, although evidence of fibrosis in the form of a reticular shadow, bronchiectasis, band-like shadow, or honeycombing was seen in 20 out of 22 (90.9%) patients. Clinco-radiological follow-up of COVID-19 by You et al. [10] found GGO in 73% and pulmonary fibrosis in 26% after a mean of 40 days after discharge from hospital. European Respiratory Society (ESR) prospective observational study showed lung abnormalities on CT in 88% at six weeks which was reduced to 56% after 12 weeks [11]. Another Chinese study of 55 patients, found 71% of patients had residual lung opacity at three months [12].

The high incidence of residual lung abnormality in our study could be due to selective inclusion of severe disease and CT performed in early follow-up. In contrast to our finding, a study by Liu C et al. [13] who included 51 COVID-19 patients with mild to severe disease showed that 2/3rd of radiological opacity subsided after four weeks of discharge. We found 90% of patients have some fibrotic-like changes at a mean of 2.5 months of follow-up which is high compared to the study by Han X et al. [14] who included similar severe COVID-19 patients with longer follow-up and at six months CT showed fibrotic-like shadow in 35%.

What these indicate is that almost all severe COVID-19 patients have some residual lung opacity present at 2-3 months and they resolve during a longer course. Pan et al. [15], in a series of 209 cases, demonstrated resolution of radiographic opacities in 75% of COVID-19 cases at one year. However, our study deals specifically with a cohort of severe COVID-19 cases, and there is an urgent need to address this issue of persistence or disappearance of fibrotic-like findings with a long prospective cohort study in this subgroup.

All post-COVID sequelae should not be labelled as lung fibrosis as it denotes permanent and irreversible damage or scar formation in the lungs, and it is one of the markers of worse prognosis [16]. So we avoid using words such as post-COVID lung fibrosis, rather fibrotic-like opacity is preferable because even fibrotic-like changes such as reticular shadow, parenchymal band, traction bronchiectasis get reduced during follow-up (Figures 2-3) [13].

Not all viral types of pneumonia are associated with residual lung opacity post-recovery, but these have been reported in 22% survivors of influenza H7N9, 33% of MERS, and 38% of SARS after a mean time interval of 6, 2-8, and 6 months, respectively [17-18]. It is no surprise that these will be seen after SARS-Cov2 also, and looking at the percentage affection with other viruses, probably the incidence with SARS-Cov2 is also no worse.

All the patients in the present series presented with some complaints and all were clinically symptomatic. Dyspnea on exertion was the most common symptom followed by cough and fatigue. A study among Italian COVID-19 survivors reported 87% having a persistent symptom after a mean follow-up of two months [2]. However, the clinical symptoms and the radiological findings do not completely match. In fact, the CT severity score may not be a good marker to assess the progress of radiological opacity. It is best used at the initial stages of the disease, when only GGOs are seen, and not later when reticular opacities, volume loss, band-formation, consolidation, and bronchiectasis develop. Further, as it takes into account the approximate volume of parenchymal involvement, the score as such does not change significantly, rather the initial changes manifest as decrease in the radiological density, and the CT severity score may persist as such. Hence there is a need to have another grading or scoring system taking into account the type of radiological findings, volume of involvement, density as well as clinical findings, when dealing with patients in the follow-up clinic.

**Figure 6:**
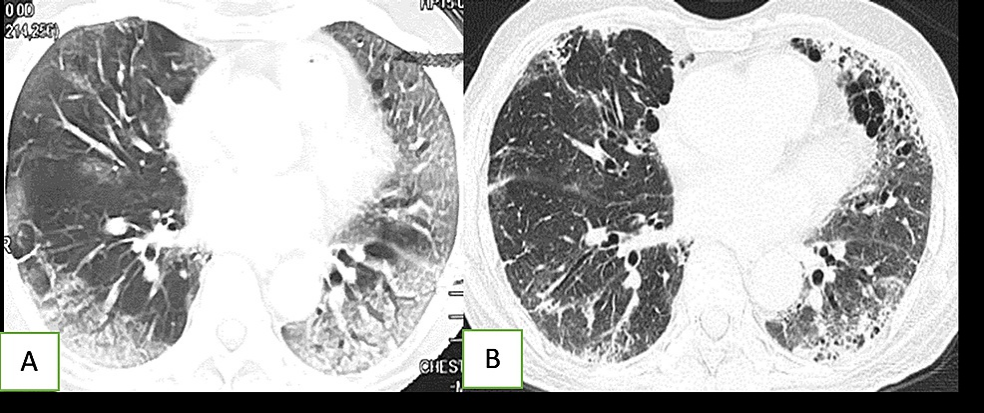
A 76-year-old man with severe COVID-19 Initial CT at presentation shows bilateral ground-glass opacities (GGOs) with peripheral predominance (A). Follow-up CT after 2.5 months shows resolution of the GGOs, and the involved areas are now showing cystic changes and some linear opacities; note that both scans have mild prominence of bronchi, which show a ‘wavy’ contour. In the second image, the cysts are not of honeycomb lung that is classical of fibrosis but can be traced to bronchial tree, however, the presence of some GGOs next to the cysts gives it the appearance of typical honeycomb lung as seen in the fibrosing conditions such as usual interstitial pneumonia (B).

Further, it is not yet clear as to the frequency and interval between imaging to assess the radiological resolution. The British Thoracic Society recommends a 12-week follow-up to provide adequate time for lung abnormalities to get resolved [17]. However, it is now well-clear that residual pulmonary opacity is present in most of the severe COVID-19 patients even up to 10-12 weeks of discharge from acute events. Fibrotic-like lesions are present in almost all patients but will likely get resolved with time (Figure 6).

True fibrosis markers, such as honeycombing, are rarely seen. Also, the bronchial and bronchiolar dilatation seen in COVID-19 usually resolves and should not be labelled as ‘traction bronchiectasis’. It is too early to conclude but we can say that the post-COVID lung sequelae including both fibrotic-like and non-fibrotic changes resolve with time and they are non-progressive as compared to true fibrotic lung diseases like idiopathic pulmonary fibrosis.

Our study has a few limitations. Firstly, only 22 patients were enrolled in the study. A bigger sample size is required to study the lung sequelae of COVID-19. Another limitation is we did not correlate with pulmonary function tests which could give more relevance to the radiological lung sequelae with lung function. To know the fate of these fibrotic sequelae, require longer follow-up, which could not be performed due to loss of follow-up. We could not include clinical details of the acute phase that may help in the identification of predictors of fibrotic-like sequelae.

## Conclusions

This study concluded that most of the severe COVID-19 patients have radiological lung sequelae during medium-term follow-up. Fibrotic-like lesions are present in almost all such patients either as the dominant feature or along with non-fibrotic findings. However, true fibrotic lesions like honeycombing are rarely seen. There is a need for long-term prospective cohort studies to know the fate of fibrotic-like lung sequelae. Every residual lung lesion should not be labelled as post-COVID lung fibrosis.

## References

[1] Fernández-de-Las-Peñas C, Palacios-Ceña D, Gómez-Mayordomo V (2021). Prevalence of post-COVID-19 symptoms in hospitalized and non-hospitalized COVID-19 survivors: a systematic review and meta-analysis. Eur J Intern Med.

[2] Carfì A, Bernabei R, Landi F (2020). Persistent symptoms in patients after acute COVID-19. JAMA.

[3] Rai DK (2020). Post-COVID-19 sequelae-issue which remain unanswered. J Appl Sci Clin Pract.

[4] Mo X, Jian W, Su Z (2020). Abnormal pulmonary function in COVID-19 patients at time of hospital discharge. Eur Respir J.

[5] Das KM, Lee EY, Singh R (2017). Follow-up chest radiographic findings in patients with MERS-CoV after recovery. Indian J Radiol Imaging.

[6] Das KM, Lee EY, Langer RD, Larsson SG (2016). Middle East respiratory syndrome coronavirus: what does a radiologist need to know?. AJR Am J Roentgenol.

[7] Hansell DM, Bankier AA, MacMahon H, McLoud TC, Müller NL, Remy J (2008). Fleischner Society: glossary of terms for thoracic imaging. Radiology.

[8] Garg M, Gupta P, Maralakunte M (2021). Diagnostic accuracy of CT and radiographic findings for novel coronavirus 2019 pneumonia: systematic review and meta-analysis. Clin Imaging.

[9] Rai DK, Sharma P, Kumar R (2021). Post covid 19 pulmonary fibrosis. Is it real threat?. Indian J Tuberc.

[10] You J, Zhang L, Ni-Jia-Ti MY (2020). Anormal pulmonary function and residual CT abnormalities in rehabilitating COVID-19 patients after discharge. J Infect.

[11] Parry AH, Wani AH, Shah NN, Jehangir M (2021). Medium-term chest computed tomography (CT) follow-up of COVID-19 pneumonia patients after recovery to assess the rate of resolution and determine the potential predictors of persistent lung changes. Egypt J Radiol Nucl Med.

[12] Zhao YM, Shang YM, Song WB (2020). Follow-up study of the pulmonary function and related physiological characteristics of COVID-19 survivors three months after recovery. EClinicalMedicine.

[13] Liu C, Ye L, Xia R (2020). Chest computed tomography and clinical follow-up of discharged patients with COVID-19 in Wenzhou City, Zhejiang, China. Ann Am Thorac Soc.

[14] Han X, Fan Y, Alwalid O (2021). Six-month follow-up chest CT findings after severe COVID-19 pneumonia. Radiology.

[15] Pan F, Yang L, Liang B (2021). Chest CT patterns from diagnosis to 1 year of follow-up in COVID-19. Radiology.

[16] Garg M, Maralakunte M, Dhooria S (2021). Sequelae of COVID-19 pneumonia: Is it correct to label everything as post-COVID lung fibrosis?. J Postgrad Med.

[17] Wang Q, Jiang H, Xie Y (2020). Long-term clinical prognosis of human infections with avian influenza A(H7N9) viruses in China after hospitalization. EClinicalMedicine.

[18] George PM, Barratt SL, Condliffe R (2020). Respiratory follow-up of patients with COVID-19 pneumonia. Thorax.

